# Pericardial Effusion in a Patient with Non-ST-Elevation Myocardial Infarction: Beware of a Hidden Malefactor

**DOI:** 10.1155/2013/365623

**Published:** 2013-03-31

**Authors:** Mamatha Punjee Raja Rao, Prashanth Panduranga, Mahmood Al-Jufaili

**Affiliations:** ^1^Department of Emergency Medicine, Royal Hospital, P.O. Box 1331, Muscat 111, Oman; ^2^Department of Cardiology, Royal Hospital, P.O. Box 1331, Muscat 111, Oman

## Abstract

Pericarditis with pericardial effusion in acute coronary syndrome is seen in patients with ST-elevation myocardial infarction specifically when infarction is anterior, extensive, and Q wave. It is very uncommon to have pericardial effusion in a patient with non-ST-elevation myocardial infarction. We present an elderly hypertensive patient who was diagnosed as non-ST-elevation myocardial infarction with pericardial effusion that turned out to be acute aortic dissection with catastrophic end. We conclude that, in patients with suspected diagnosis of non-ST-elevation myocardial infarction or unstable angina, if pericardial effusion is detected on echocardiography, aortic dissection needs to be considered.

## 1. Background

Pericarditis with pericardial effusion (PE) in acute coronary syndrome is seen in patients with ST-elevation myocardial infarction (MI) specifically when infarction is anterior, extensive, and Q wave [1–3]. It is very uncommon to have PE in a patient with non-ST-elevation MI. We present an elderly hypertensive patient who was diagnosed as non-ST-elevation myocardial infarction with pericardial effusion that turned out to be acute aortic dissection with catastrophic end.

## 2. Case Presentation

**Figure 1:**
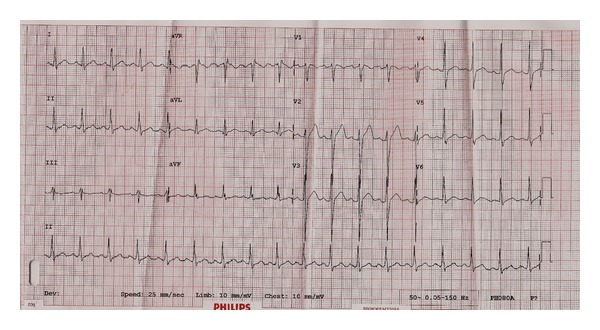
12-lead electrocardiogram showing sinus rhythm with left ventricular hypertrophy and no significant acute ischemic changes in a patient with ascending aortic dissection.

A 50-year-old obese woman with history of hypertension was diagnosed to have non-ST-elevation MI. She visited a regional health center with very severe central chest pain and profuse sweating lasting for 30 minutes. Blood pressure was noted to be 90/60 mmHg, and pulse rate was 100 beats per minute. ECG was reported to show evidence of left ventricular (LV) hypertrophy with minimal inferolateral ST depression. Her troponin T was 0.023 *μ*g/L (normal <0.010 *μ*g/L), and on repeating, it jumped to 6.16 *μ*g/L. A chest X-ray was done and was interpreted to be normal. D-dimer level was not done. Transthoracic echocardiogram done there reported concentric LV hypertrophy with hypokinesia of septum and lateral wall with good LV systolic function. There was mild PE. She was treated with small dose of dobutamine for few hours along with morphine, antiplatelets, and anticoagulants. She was transferred next day to our institute for percutaneous coronary intervention. On arrival in emergency department, she was hemodynamically stable, off inotropes with no pain, and all peripheral pulses intact. Blood pressure was 150/70 mmHg, and pulse rate was 70 beats per minute. Cardiac examination revealed normal jugular venous pressure, no ankle edema, and no gallop, but there was a harsh ejection systolic murmur in aortic area. Repeat ECG was nondiagnostic for acute coronary syndrome (Figure 1). Hence an urgent repeat transthoracic echocardiogram was performed to confirm regional wall motion abnormalities and PE as well as to rule out aortic dissection in view of severe chest pain at the onset and transient unexplained hypotension.

**Figure 2:**
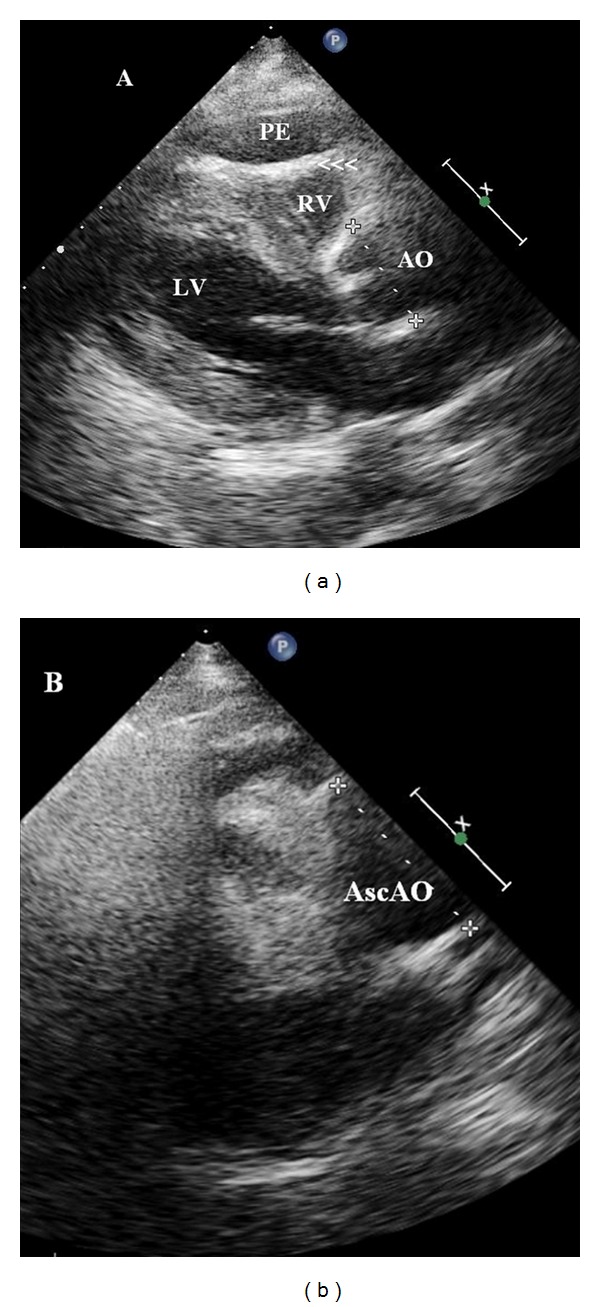
Transthoracic echocardiogram in parasternal long axis view showing moderate pericardial effusion with diastolic collapse of right ventricle (A, arrowheads) in a patient with ascending aortic dissection. Note markedly dilated ascending aorta (B, 5.9 cm). LV: left ventricle; RV: right ventricle; AO: aorta; PE: pericardial effusion; AscAo: ascending aorta.

**Figure 3:**
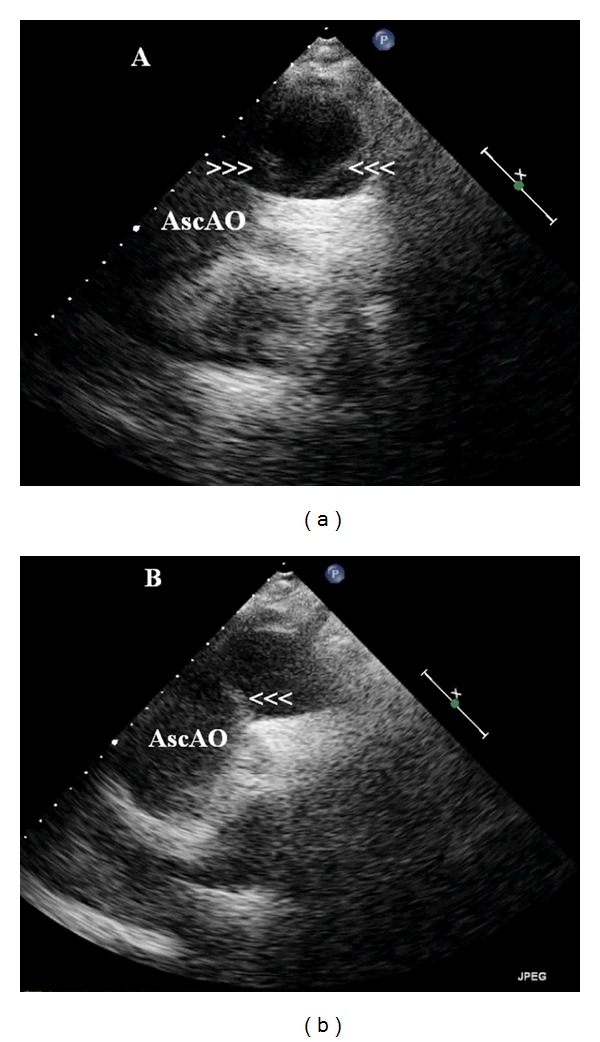
Transthoracic echocardiogram in suprasternal view showing dissection flap in ascending aorta extending up to arch dividing the aorta into a large false lumen and a small true lumen (arrowheads) in a patient with ascending aortic dissection. AscAo: ascending aorta.

Transthoracic echocardiogram showed mildly dilated LV and atrium with moderate concentric LV hypertrophy and no significant regional wall motion abnormalities. Right side was not dilated. However, there was moderate circumferential PE (19 mm) with evidence of early right atrial and ventricular diastolic collapse (Figure 2(a), arrowheads). There was mild aortic, mitral, and tricuspid regurgitation. Aortic valve was sclerotic with no stenosis. Aortic root and ascending aorta were dilated. Aortic annulus measured 3.2 cm, aortic root 4.1 cm, and ascending aorta 5.9 cm (Figure 2(b), arrowheads). There was no evidence of dissection flap in aortic root or proximal ascending aorta. Immediately, a suprasternal view was obtained, which showed a long freely mobile dissection flap in distal ascending aorta up to the arch (Figures 3(a) and 3(b), arrowheads). Immediately, patient was seen by a cardiac surgeon, and operating room was alerted. As patient was hemodynamically stable, cardiac surgeon arranged for an emergency computed tomographic (CT) scan to know the exact extent of the dissection. Patient was shifted to CT room. However, patient was arrested on CT table prior to the scan. Patient went into pulseless electrical activity, and inspite of resuscitative measures including 50 mL hemorrhagic pericardiocentesis, she expired. 

## 3. Discussion

The main causes for PE post-ST-elevation MI are early pericarditis with effusion due to bleeding from a transmural infarcted myocardial wall, in addition to heart failure [4, 5]. PE in non-ST-elevation MI in the absence of heart failure is uncommon due to presence of a nontransmural infarct. On the contrary, in one-third of patients with ascending aortic dissection (AAD), the thin walled false lumen may rupture into the pericardium causing PE with or without tamponade [5, 6]. Pericarditis may be the first and only presentation of AAD [7]. In this patient, the presence of PE in a diagnosis of non-ST-elevation MI should have aroused the suspicion of other causes for chest pain rather than acute coronary syndrome. 

Since aortic dissection symptoms mimic other more common conditions, a high index of clinical suspicion is needed to diagnose AAD [6, 8]. About 90% of acute dissections present with acute pain in the chest, the back, or both [8]. However, the dissection pain differs from coronary pain by being most severe at its onset in contrast to the less intense, crescendo-like pain of unstable angina or MI [8]. In addition, the pain may migrate as the dissection progresses, or it may subside, misleading the physicians [6, 8]. This was typically seen in this patient. This patient had severe chest pain at onset which subsided later. The classic presentation of acute, severe chest or back pain associated with accelerated hypertension and signs of aortic regurgitation along with pulse deficits is not seen most of the time [9]. Significant aortic regurgitation may not occur if the dissection does not involve aortic root or cusps as noted in this patient with dissection flap noted in distal ascending aorta. Many patients with type A AAD present with normal blood pressure or hypotension in comparison to type B dissection patients who are hypertensive on presentation [6]. This was similar in this patient with initial hypotension. Pulse deficits and normal chest X-ray occur in up to 30% and 15% of AAD, respectively [6, 8]. D-dimer levels rise in acute aortic dissection within 24 hours of chest pain but cannot rule out aortic dissection in high risk individuals [6, 8]. However, D-dimer was not done in the first hospital.

Furthermore, it is known that dissection flap can cause ischemia or infarction due to malperfusion of a coronary artery (commonly right coronary artery) in 1% to 7% of AAD [6]. Additionally, myocardial ischemia in the setting of dissection may lead to a delay in the diagnosis of dissection and to bleeding complications from antiplatelet and anticoagulant drugs given to treat the acute coronary syndrome [8]. This was also seen in this patient with initial diagnosis of acute coronary syndrome as well as increase in PE from mild to moderate. It has been noted that cardiac tamponade, occurring in about 10% of AAD, signals a higher risk of death as it happened in this patient [6, 8]. It is well known that in patients with aortic dissection and cardiac tamponade, routine pericardial drainage is contraindicated (must go for surgery) as it may increase the leak or rupture by restoring blood pressure, which then increases the tear and the driving pressure of the leak, thereby increasing mortality [10]. In the recent American guidelines, limited pericardiocentesis is advised to restore perfusion in patients with hemopericardium and cardiac tamponade who cannot survive until surgery or if the patient in cardiac arrest [6].

This case reiterates that not all aortic dissection cases present classically, and a high index of suspicion is needed to diagnose aortic dissection. Furthermore, in patients with acute coronary syndrome, PE is common in patients with ST-elevation MI, and this should not be extrapolated to patients with non-ST-elevation MI or unstable angina. We conclude that, in patients with diagnosis of non-ST-elevation MI or unstable angina, if PE is detected on echocardiography, aortic dissection needs to be considered. Such patients need to undergo urgent CT to confirm or rule out aortic dissection.

## Supplementary Material

Transthoracic echocardiogram in suprasternal view showing a mobile dissection flap in ascending aorta in a patient with diagnosis of non-ST-elevation myocardial infarction.
